# Right Atrial Thrombus Presenting as Platypnea-Orthodeoxia Secondary to Reverse Lutembacher Syndrome: A Case Report

**DOI:** 10.7759/cureus.26754

**Published:** 2022-07-11

**Authors:** Khizar Hamid, Swaminathan Perinkulam Sathyanarayanan, Kayla Hoerschgen, Mohammad Ali, John C Yu

**Affiliations:** 1 Internal Medicine, University of South Dakota Sanford School of Medicine, Sioux Falls, USA; 2 Pathology, University of South Dakota Sanford School of Medicine, Sioux Falls, USA; 3 Pulmonary and Critical Care Medicine, Sanford University of South Dakota (USD) Medical Center, Sioux Falls, USA

**Keywords:** patent foramen ovale, impella device, tricuspid valve stenosis, coronary arterial fistula, platypnea-orthodeoxia syndrome, atrium thrombus, right atrial myxoma

## Abstract

Platypnea-orthodeoxia syndrome (POS) is defined by dyspnea and deoxygenation due to a change in body position from lying down to an upright position. We present a case of a large right atrial (RA) thrombus likely due to a right coronary artery fistula in a patient with a patent foramen ovale (PFO). On imaging, the thrombus was thought to be an atrial myxoma involving the tricuspid valve; however, after surgical excision and histopathological analysis, it was noted to be a cystic thrombus. Red-brown material along with vascular elements was noted on histopathology. Post-surgery, the patient was critically ill and died due to severe tricuspid regurgitation (TR) and hypotension despite using a right ventricle assist device and multiple vasopressors. Reverse Lutembacher syndrome (RLS) is defined as a triad of tricuspid stenosis (TS), elevated RA pressure, and right-to-left atrial shunting. The location of the mass and positional changes could be causing transient RLS from positional TS and interatrial shunting via the PFO causing POS. Cardiac magnetic resonance imaging can help differentiate between intracardiac masses. T1 and T2 signal characteristics and differences in contrast enhancement can help differentiate between a thrombus and a tumor. Treatment options include anticoagulation, thrombolysis, and thrombectomy. If severe TR occurs after surgery, treatment modalities such as caval valves could be an option in the future. Extracorporeal membrane oxygenation to provide right ventricle support in such cases could be considered.

## Introduction

Platypnea-orthodeoxia syndrome (POS) is an uncommon condition characterized by dyspnea and deoxygenation due to a change from a recumbent to a sitting or standing position [1]. It is defined as a decrease in arterial oxygen saturation (SaO2) > 5% or partial pressure of oxygen (PaO2) > 4 mmHg [2]. The first case of POS was described by Burchell et al. in 1949 in a patient with post-traumatic intrathoracic venous-arterial shunting [3]. The exact mechanism of POS is unknown; however, it is associated with cardiac, pulmonary, abdominal, and autonomic dysfunctions [3]. Commonly it is seen if there is right-to-left interatrial shunting in the setting of patent foramen ovale (PFO), atrial septal defect, or atrial septal aneurysm, which eventually leads to spontaneous or induced pulmonary hypertension [3].

## Case presentation

An 80-year-old man with a past medical history of hypertension, prostate cancer treated with radiation, and hypothyroidism presented to the hospital with complaints of shortness of breath. He had been hypoxic five months before the presentation at a clinic visit, but no intervention was pursued. Three months before the presentation, during a routine screening colonoscopy, he was found to be transiently hypoxic leading to the procedure being aborted. He was seen by the pulmonary clinic two months before the presentation and no formal diagnosis was made, but oxygen supplementation was initiated. He had a progressive decline in his energy but denied having any chest pain, cough, fever, or dyspnea with exertion. More dyspnea was noted in the upright position, which was relieved by lying flat. POS was confirmed at the presentation. Vital signs were temperature of 98.5°F, blood pressure of 133/85 mmHg, pulse rate of 74 beats/minute, respiratory rate of 16 breaths/minute, and oxygen saturation of 94% on 4 L of oxygen via nasal cannula. Physical examination was unremarkable. Labs at presentation were unremarkable apart from mild hypoxemia and slight thrombocytopenia (Table 1).

**Table 1:** Labs at presentation showing, hypoxia, hypocapnia, and minor thrombocytopenia. pCO2: partial pressure of carbon dioxide; pO2: partial pressure of oxygen.

Labs (reference range)	Value
Arterial blood gas (pH: 7.35-7.45; pCO2: 35-45 mmHg; pO2: 80-104 mmHg)	pH: 7.45; pCO2: 31 mmHg; pO2: 61 mmHg
Hemoglobin (11.5-15.8 g/dL)	14.8 g/dL
White blood cell count (4-11 K/uL)	4.7 K/uL
Platelet count (140-400 K/uL)	137 K/uL
Sodium (136-145 meq/L)	138 meq/L
Potassium (3.5-5.1 meq/L)	4.1 meq/L
Calcium (8.5-10.5 mg/dL)	8.8 mg/dL
Chloride (98-109 meq/L)	107 meq/L
Bicarbonate (22-31 meq/L)	25 meq/L
Blood urea nitrogen (10-25)	18 mg/dL
Creatinine (0.55-1.02 mg/dL)	0.78 mg/dL

**Figure 1:**
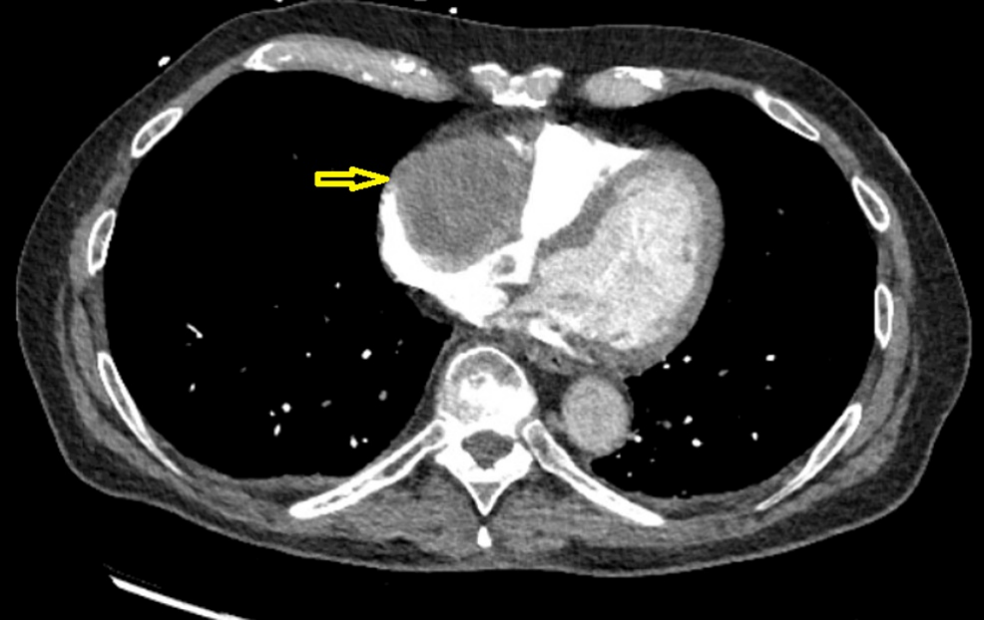
Computed tomography angiogram for pulmonary embolism. Yellow arrow pointing to contrast filling defect at the location of the right atrial thrombus.

**Figure 2:**
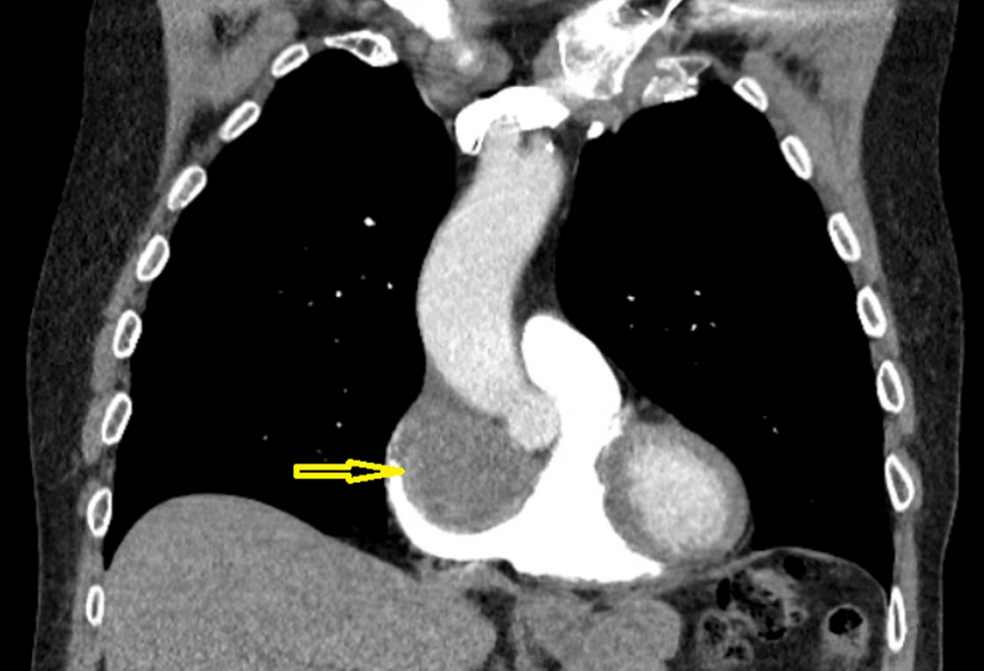
Computed tomography angiogram for pulmonary embolism. Yellow arrow pointing to contrast filling defect at the location of the right atrial thrombus.

Computed tomography (CT) of the chest revealed a right atrial (RA) mass. This was followed by a CT angiogram (CTA) of the chest, which excluded pulmonary embolism (PE) and other lung pathology but revealed a 4.7 x 5.6 x 4.3 cm RA mass, suspicious for a cardiac myxoma (Figures 1, 2).

**Figure 3:**
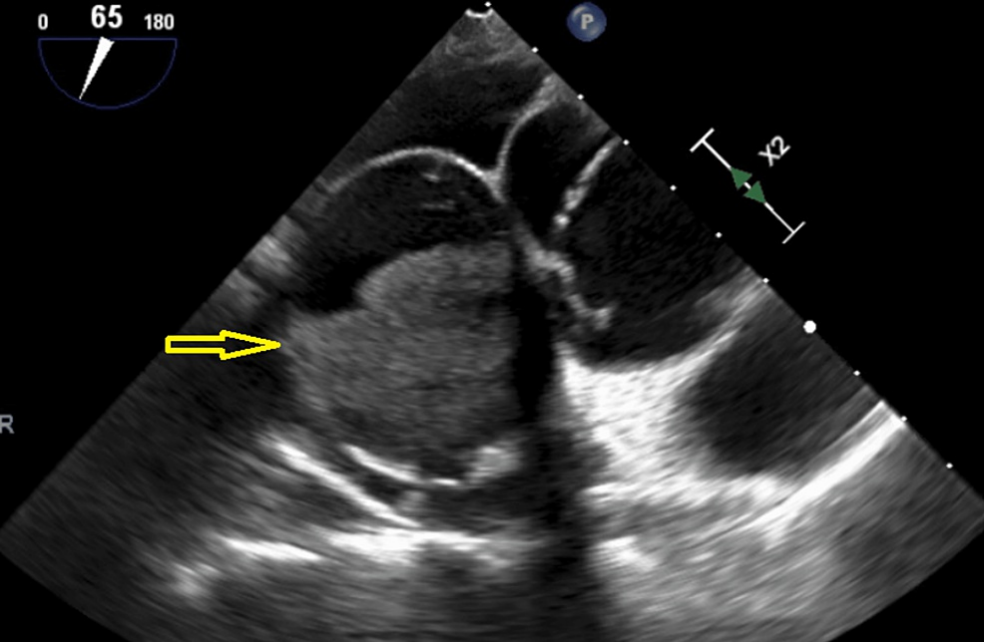
Transthoracic echocardiogram. Yellow arrow showing right atrial mass.

Subsequently, a transthoracic echocardiogram and a transesophageal echocardiogram (TEE) were done to better characterize it. TEE revealed a left ventricular (LV) ejection fraction of 50-55% with no wall motion abnormalities. The left atrium (LA) was mildly dilated, the right ventricle (RV) was normal in size and systolic function, and RA was normal in size but revealed a large mass measuring 5.9 x 3.8 cm, localized anteromedially (Figure 3).

**Figure 4:**
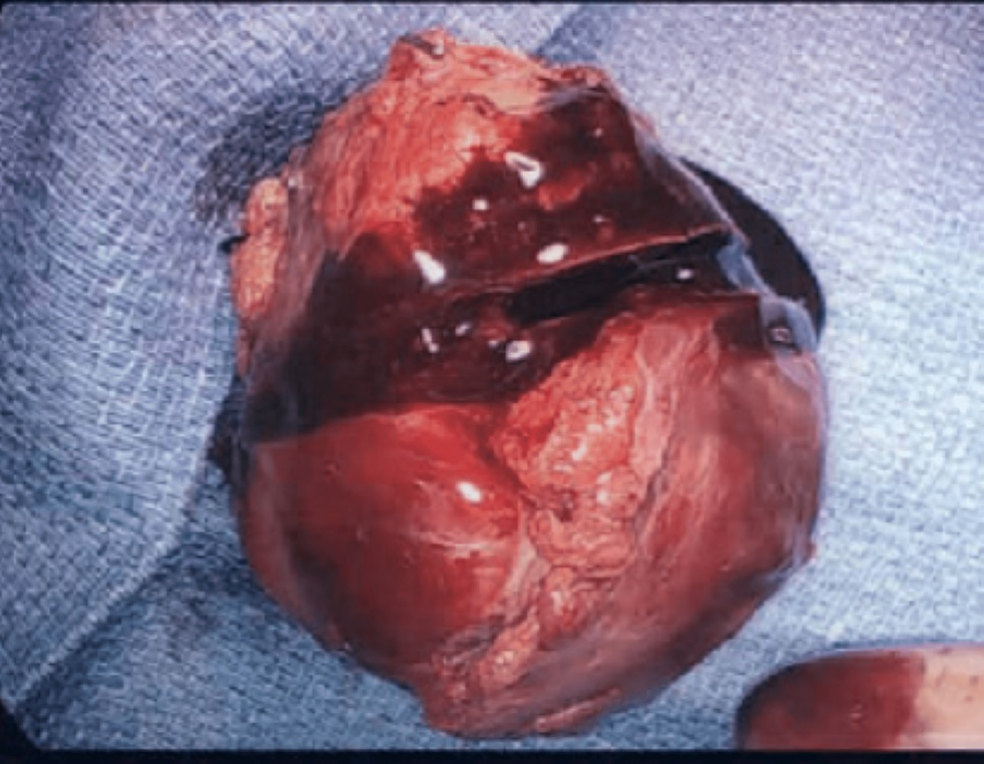
Resected right atrial mass (7.7 x 5.9 x 4.2 cm red-pink mass). The specimen is sectioned to reveal it to be entirely replaced by a cyst, which contains a red-brown, friable material that is loosely attached to the smooth-lined wall.

Mild tricuspid regurgitation (TR) by color flow Doppler was noted and the atrial mass was directly adjacent and appeared to be adherent to the tricuspid valve (TV) cusp. A PFO was also visualized. Cardiothoracic surgery (CTS) was consulted for mass resection. The patient underwent left and right heart catheterization before CTS and a severely ectatic, severely tortuous, and severely diffuse right coronary artery (RCA) disease was noted. CTS revealed the mass had involved the anterior leaflet of the TV, which was thickened and sclerotic. It was giving an aberrant arterial supply to the distal RCA. It was resected along with most of the anterior aspect of the tricuspid annulus (TA) (Figure 4).

**Figure 5:**
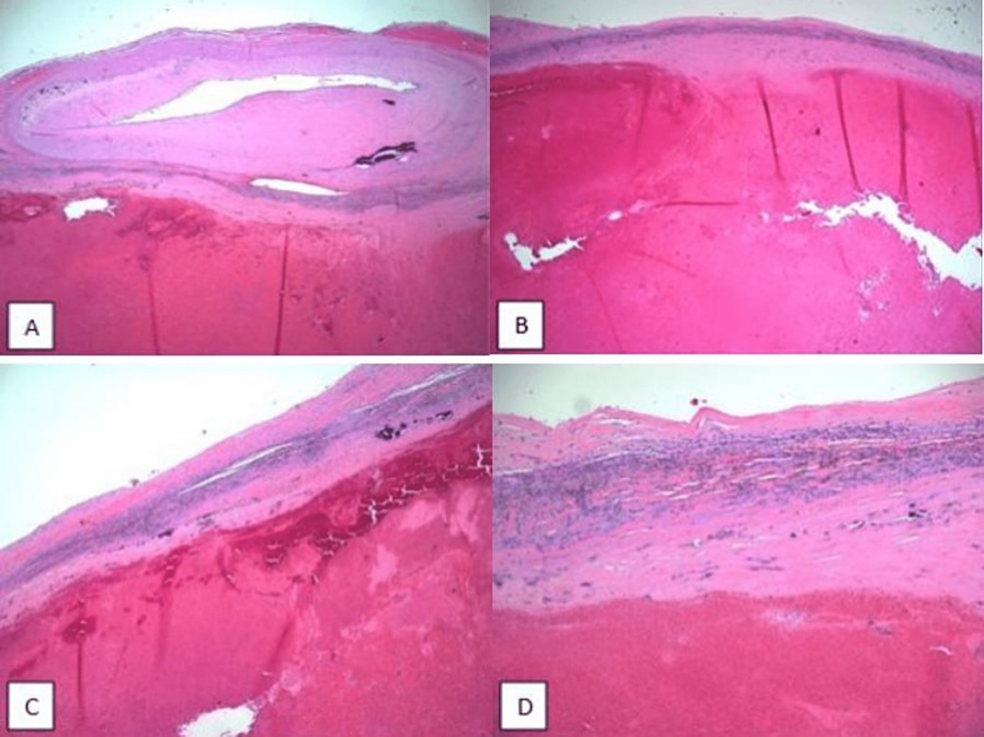
Right atrial mass excision. (A) A vascular segment with moderate calcific atherosclerosis and an adjacent large organizing thrombus (hematoxylin and eosin (H&E), 2x magnification). (B) A large organizing thrombus with a fibrous wall (H&E, 2x magnification). (C) A large organizing thrombus and fibrotic wall with chronic inflammation and focal calcification (H&E, 4x magnification). (D) Fibrotic wall with chronic inflammation, hemosiderin deposition, and focal calcification (H&E, 10x magnification).

Endoscopically harvested right saphenous vein graft was used for coronary artery bypass to the distal RCA. A moderate-sized PFO was noted, which was repaired. The RA was then reconstructed using autologous pericardium. TV replacement was not possible as most of the anterior aspect of the TA was resected with the mass. TEE performed after coming off cardioplegic bypass revealed severe TR. Pathological evaluation of the mass revealed a large organizing thrombus with a fibrous wall showing focal calcifications and an adjacent vascular segment with moderate calcific atherosclerosis. No true neoplasm was identified raising the possibility of a prior aneurysm or other vascular malformation with secondary hematoma (Figure 5).

The patient was extubated to bilevel positive airway pressure; however, he continued to be hypotensive requiring four vasopressors to maintain mean arterial pressure (MAP) greater than 65 mmHg. He developed severe right heart failure requiring the placement of an Impella right ventricular assist device (Abiomed, Danvers, MA). Despite these measures, multiorgan failure developed requiring reintubation. A family meeting was convened and the patient was transitioned to comfort care and passed away.

## Discussion

The patient presented with POS secondary to an atrial mass, which was a large organizing thrombus with a fibrous wall, with the possibility of prior vascular malformation and secondary hematoma (Figure 5). Some of the causes of cardiac masses include cardiac myxomas, hemangiomas, and thrombi. Initially, there was a concern for atrial myxoma, which was excluded after no true neoplasm was noted on histopathological analysis.

Cardiac myxoma is the most common benign cardiac tumor and can arise in any chamber of the heart with 75% of the cases occurring in the LA [4]. Other sites include the RA, RV, and LV in descending order of occurrence [5]. Rare cases of RA myxomas leading to RV inflow obstruction causing dyspnea have been reported in the literature [5,6]. In contrast, atrial hemangiomas are rare and account for 2.8% of all cardiac tumors and most are in the RA [7]. Due to its rarity, it has a high preoperative misdiagnosis rate and is confirmed after surgery and histopathological analysis [8]. Our patient had a right atrial thrombus (RAT) that was involving the anterior leaflet of the TV causing RV inflow obstruction.

Reverse Lutembacher syndrome defined as a triad of TV stenosis, elevated RA pressure, and right-to-left shunt could also be at play [9]. Positional changes of the thrombus leading to transient TV stenosis together with the PFO could be causing temporary right-to-left shunting and ventilation-perfusion mismatch, as evidenced by dilated LA in our patient [10]. Holcman et al. described a similar presentation; however, their patient had elevated right-sided pressures and had Hodgkin's lymphoma contributing to the hypercoagulable state [11]. Vargas-Beal et al. described the first case of RAT causing POS via a right-to-left shunt and that patient had protein C deficiency, another hypercoagulable condition [12]. The exact prevalence of RAT is unknown but it was seen in 7% of 23,796 autopsies conducted in one review [13]. Syncopal episodes due to RAT have also been reported [14]. The presence of a foreign body such as a central venous catheter can lead to the development of RAT and subsequent PE [15]. Occasionally presumed thrombi do not respond to treatment with anticoagulation and can be confused with tumors [16]. Our patient did not have any catheters or a known hypercoagulable state. The cystic nature of the mass containing red-brown friable material (Figure 4) and the histological analysis showing a vascular segment (Figure 5) could indicate this cyst containing a thrombus could be due to a right coronary artery fistula. A very similar presentation is described by Wen et al., in which a right heart mass suspected to be a myxoma was found to be a right coronary artery fistula during CTS; in that scenario, no cardiac magnetic resonance (CMR) imaging was done as well [17].

CMR is a useful accurate tool to differentiate cardiac masses and can be incorporated into routine evaluation [18]. In atrial myxoma, heterogenous intermediate T1 and heterogenous hyperintense T2 signals are noted. In contrast, atrial thrombus has a homogenous hypointense T1 and T2 signal in a chronic thrombus. Signal return is dependent on the acute, subacute, and chronic nature of the thrombus. Gadolinium enhancement has a heterogonous pattern in myxoma compared to lack of enhancement in a thrombus, unless a high level of fibrous tissue is present [19]. Patients with suspected thrombus can be treated with anticoagulation, thrombolysis, and thrombectomy [13]. If the thrombus involves the TV, removal of it can lead to severe TR. Newer treatment methods, such as caval valve implantation (CAVI), which do not utilize the TA could be considered in these cases as they have been shown to improve hemodynamics [17]. CAVI can be performed safely in high-risk severe TR surgical populations; however, more research is needed [20]. Extracorporeal membrane oxygenation (ECMO) can be utilized to decrease RV preload and RV tension and to deliver oxygenated blood to the coronary circulation to help with recovery [21].

## Conclusions

Large RAT can present with dyspnea and POS, particularly if present near the RV inflow tract and TV. RCA fistula can cause such a thrombus even in the absence of hypercoagulability disorders or an intracardiac foreign body. It should be in the differential diagnosis. CMR can help differentiate between a tumor and a thrombus. Treatment can be with anticoagulation, thrombolysis, and thrombectomy. If a thrombus is involving the TV leaflets, there is a possibility of severe TR post-CTS surgery. ECMO capability in such a scenario can provide temporary support while definitive measures such as CAVI can be contemplated.
